# Emplacement and associated sedimentary record of the Jurassic submarine salt allochthon of the Wurzeralm (Eastern Alps, Austria)

**DOI:** 10.1111/ter.12675

**Published:** 2023-08-07

**Authors:** Maditha Kurz, Oscar Fernandez, Lino Eggerth, Bernhard Grasemann, Philipp Strauss

**Affiliations:** ^1^ Department of Geology University of Vienna Vienna Austria; ^2^ OMV Vienna Austria

**Keywords:** diapir, Eastern Alps, Fossil salt sheet, gypsum mylonite, syn‐tectonic sedimentation

## Abstract

A fossil salt sheet emplaced in the Jurassic in submarine conditions is described in the Eastern Alps of Austria, providing unique insights into the emplacement of similar submarine structures and their potential control on depositional systems. The salt sheet is a plug‐fed extrusion emplaced due to squeezing of a salt diapir under compression. The preserved mylonitic shear fabric in the evaporites indicates radial, south‐directed emplacement of the salt sheet. Tectono‐sedimentary relationships record the evolution of the salt structure, from initial diapiric growth, to salt sheet extrusion and posterior collapse. Syn‐extrusion sediments record the variable bathymetry of the extruding salt sheet, with reefal carbonates building up on the crestal bulge while their deeper water equivalents accumulated on the extruding salt lobe. This is the first description of a salt allochthon still linked to its source diapir in the Eastern Alps.

**Significance Statement**

This article presents a unique example of a preserved Jurassic‐age submarine allochthonous salt sheet and associated syn‐allochthony sediments in the Eastern Alps of Austria. Similar structures are normally only seen on reflection seismic data in the offshore, and this example provides a direct analogous structure that can be used to understand the internal structure of similar bodies and their control on syn‐emplacement sedimentation. This example stands in contrast to other allochthonous salt bodies described in the field (e.g. namakiers of Iran) whose emplacement occurs in sub‐aerial conditions. Finally, this is the first occasion a similar structure is described in the Eastern Alps and can have significant implications for the geodynamic evolution of this segment of the Alpine system in the Jurassic.

## 1 INTRODUCTION

Allochthonous salt bodies are common features of salt basins worldwide and have been abundantly documented in both convergent and passive margin settings (Hudec & Jackson, 2006; Rowan, 2017). The mechanism of emplacement of these bodies has been studied in detail in the Zagros *namakiers*, emplaced subaerially (Mansouri et al., 2019; Schleder & Urai, 2007; Závada et al., 2021). In other contexts, outcrop conditions (weathering, vegetation), tectonic overprint or inaccessibility (as in submarine examples) make similar analysis impossible.

In this paper, we present a uniquely well‐preserved example of a plug‐fed extrusion (sensu Hudec & Jackson, 2006) emplaced in a submarine setting in the Late Jurassic. The allochthonous salt sheet is located in the Northern Calcareous Alps (Eastern Alps, Austria), and has been mostly preserved in its original geometry, in spite of post‐extrusion Alpine deformation. The extruded evaporite body (made up at present mostly of gypsum) excellently preserves original extrusion shear fabrics and syn‐emplacement roof sediments. This paper summarizes the available field evidence to provide a kinematic and tectono‐sedimentary model for salt sheet extrusion in submarine settings.

## 2 GEOLOGICAL SETTING

The Northern Calcareous Alps (NCA) are a set of imbricate thrust sheets of Upper Permian to Eocene strata mostly detached from their Variscan basement (Linzer et al., 1995; Schmid et al., 2004; Wagreich & Faupl, 1994). The NCA Upper Permian to Middle Jurassic succession was deposited on Adria, a continental block forming the northern passive margin of the Neo‐Tethys (Mandl, 2000; Schmid et al., 2008). From Middle to Late Jurassic, Adria rifted and separated from Europe forming the Alpine Tethys (Mandl, 2000; Schmid et al., 2008). An early phase of Alpine orogenesis in the Late Jurassic caused initial inversion of the margin and shortening in the NCA (Fernandez et al., 2023; Frank & Schlager, 2006; Gawlick & Missoni, 2019; Ortner, 2017). From Early Cretaceous to Palaeogene times, the NCA were thrust over the Alpine Tethys and European continental margin (Linzer et al., 1995; Stüwe & Schuster, 2010).

**FIGURE 1:**
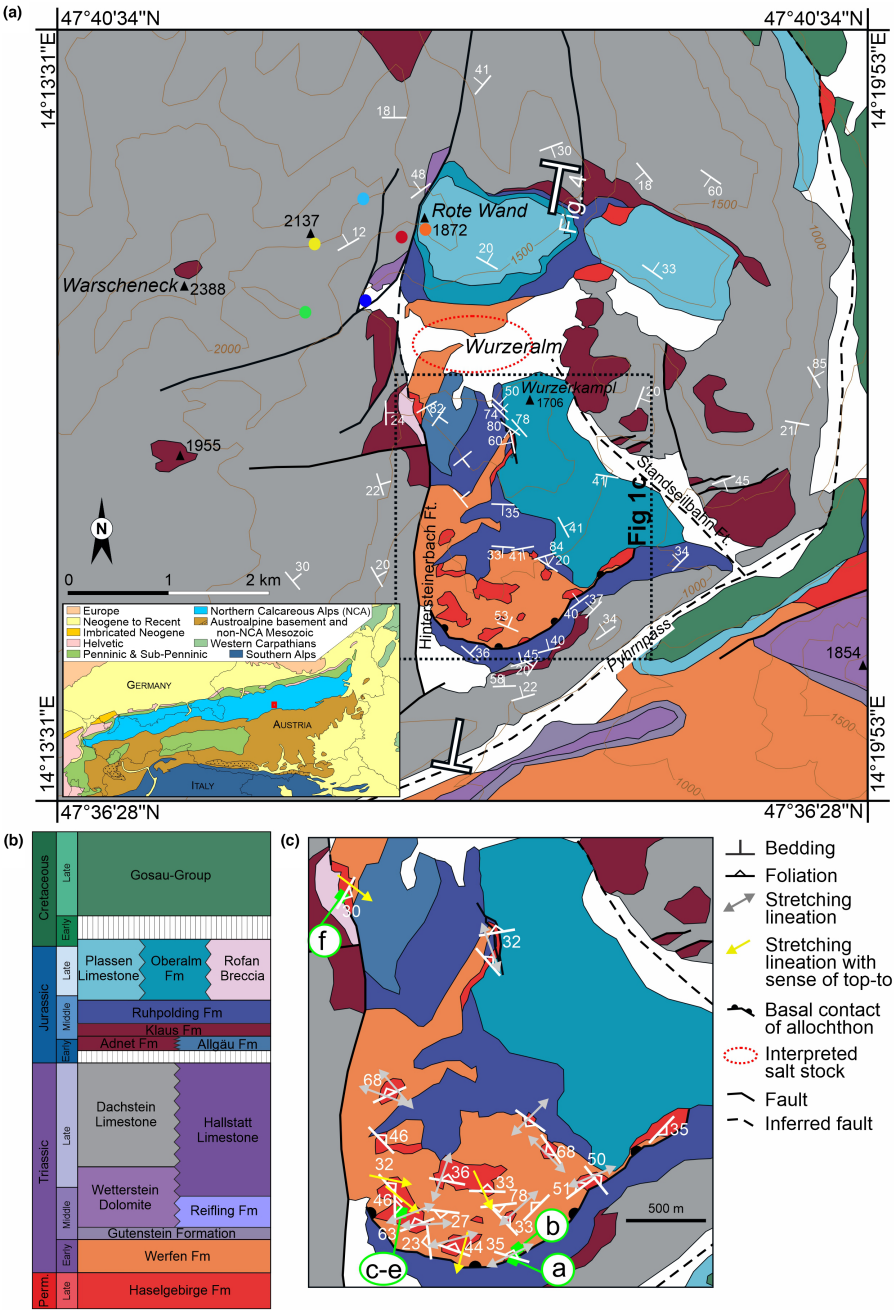
(a) Detailed geological map of the Wurzeralm and surrounding area (after Kurz, 2022; Moser & Pavlik, 2014; Ottner, 1990; and mapping by the authors) with bedding dip measurements. The colour legend follows the scheme in (b). The inset shows the location of the study area within the Eastern Alps (red box). The coloured dots in the area of the Warscheneck‐Rote Wand are points shown also in Figure 2 for location reference. The location of (c) and of the profile in Figure 4 are shown. (b) Simplified stratigraphic chart of the area roughly scaled for the relative thickness of units in the area. (c) Detail of the Wurzeralm salt sheet, south of the inferred salt stock feeder, with foliation (and their dip magnitude) and stretching lineations measured in Haselgebirge Fm gypsum. Lineations that are accompanied by shear sense indicators are shown as unidirectional arrows indicating “top‐to‐the” direction. The location of the photographs in Figure 3 is indicated by labels a‐f.

**FIGURE 2:**
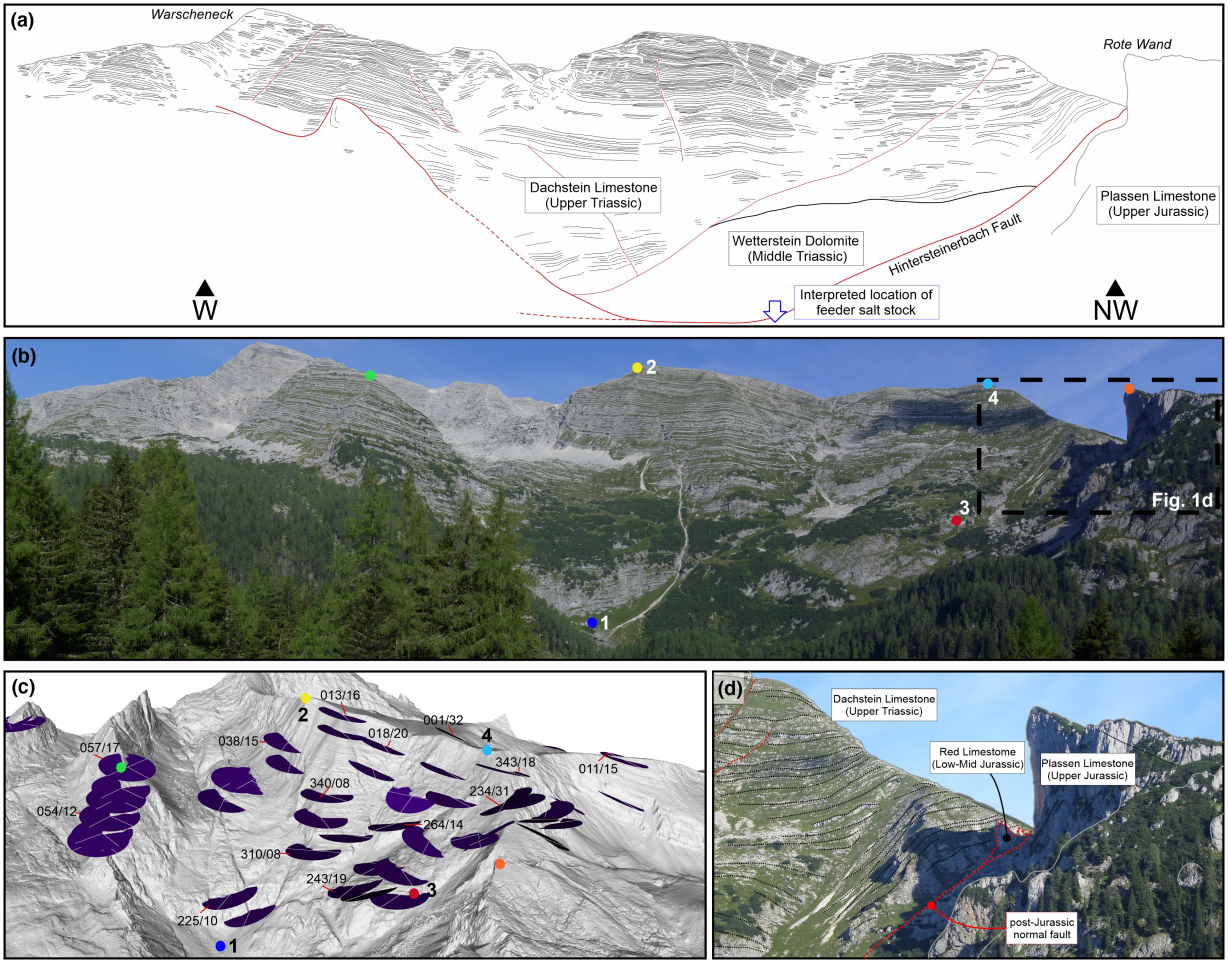
(a,b) Panorama looking west of the Wurzeralm, and line drawing showing a thinning of the Dachstein Limestone towards the Wurzeralm diapir. The Plassen Limestone of the Rote Wand sits above the collapsed Wurzeralm diapir. (c) Oblique view of a Lidar digital terrain model of the area in the panorama in (a) (digital terrain model data obtained from Land Oberösterreich ‐ data.ooe.gv.at, under CC‐BY‐4.0 licence). Blue disks represent bedding dip and show progressive rotation of the beds, from the bottom to the top of the Dachstein Limestone. Coloured dots represent reference points in (b) (shown also in Figure 1a). The stratigraphic thickness of the Dachstein Limestone reduces from 800 m between dots labelled 1 and 2, to 500 m between dots labelled 3 and 4. (d) Detail from the panorama in (a) showing a small‐scale syncline developed in the Dachstein Limestone in the proximity to its contact with the diapir.

Mandl (2000) provides an overview of the NCA stratigraphy, summarized here (Figure 1b). An initial Upper Permian red bed succession deposited during rifting is overlain by the Permo‐Triassic Haselgebirge Fm, a thick sequence of evaporites and shales (Leitner et al., 2017). These units are followed by late rift Lower Triassic epi‐continental clastics (Werfen Fm) and Middle Triassic shallow water carbonates (Gutenstein Fm). Thermal subsidence and salt‐evacuation during the Middle to Late Triassic provided accommodation space for the deposition of a 2‐ to 3‐km‐thick sequence of shallow platform carbonates (Wetterstein and Dachstein Limestones) that record syn‐depositional diapir growth in the form of rapid thickness changes and folding (Figure 2) (cf. Fernandez et al., 2021; Strauss et al., 2021). The Reifling Fm and the Hallstatt Limestone (absent in the study area) are their deep water equivalents.

Continued subsidence in combination with reduced carbonate productivity during the Early Jurassic led to the drowning of platforms and the accumulation of condensed deep water red crinoidal limestone above submarine swells (Adnet Fm) and a marly turbidite succession in intervening basins (Allgäu Fm). Middle Jurassic is also condensed stratigraphy and represented by the Mn‐nodule rich, red Klaus Fm and/or the Ruhpolding Fm radiolarites and siliceous limestones. A major change occurred in the Upper Jurassic, with the deposition of the shallow water reefal Plassen Limestone and its deeper water marly limestone equivalent (Oberalm Fm). The Upper Jurassic is also characterized by the presence of localized pockets of breccias (Rofan Breccia, sensu Ottner, 1990).

The youngest deposits in the area are syn‐orogenic clastics of the Gosau Group (Wagreich & Decker, 2001).

## 3 THE WURZERALM SALT SHEET

The Wurzeralm salt sheet is located on the trailing (southernmost) edge of the preserved central NCA thrust sheets. The Wurzeralm diapir and salt sheet outcrop define a roughly N‐S rectangular area characterized by a somewhat patchy arrangement of Jurassic units and Haselgebrige and Werfen Fms (Figure 1a), completely surrounded by the Dachstein Limestone and Adnet and Klaus Fms (Figure 1a).

West of the Wurzeralm, the Dachstein Limestone thins against the diapir, indicating syn‐depositional diapir growth (Figure 2).

**FIGURE 3:**
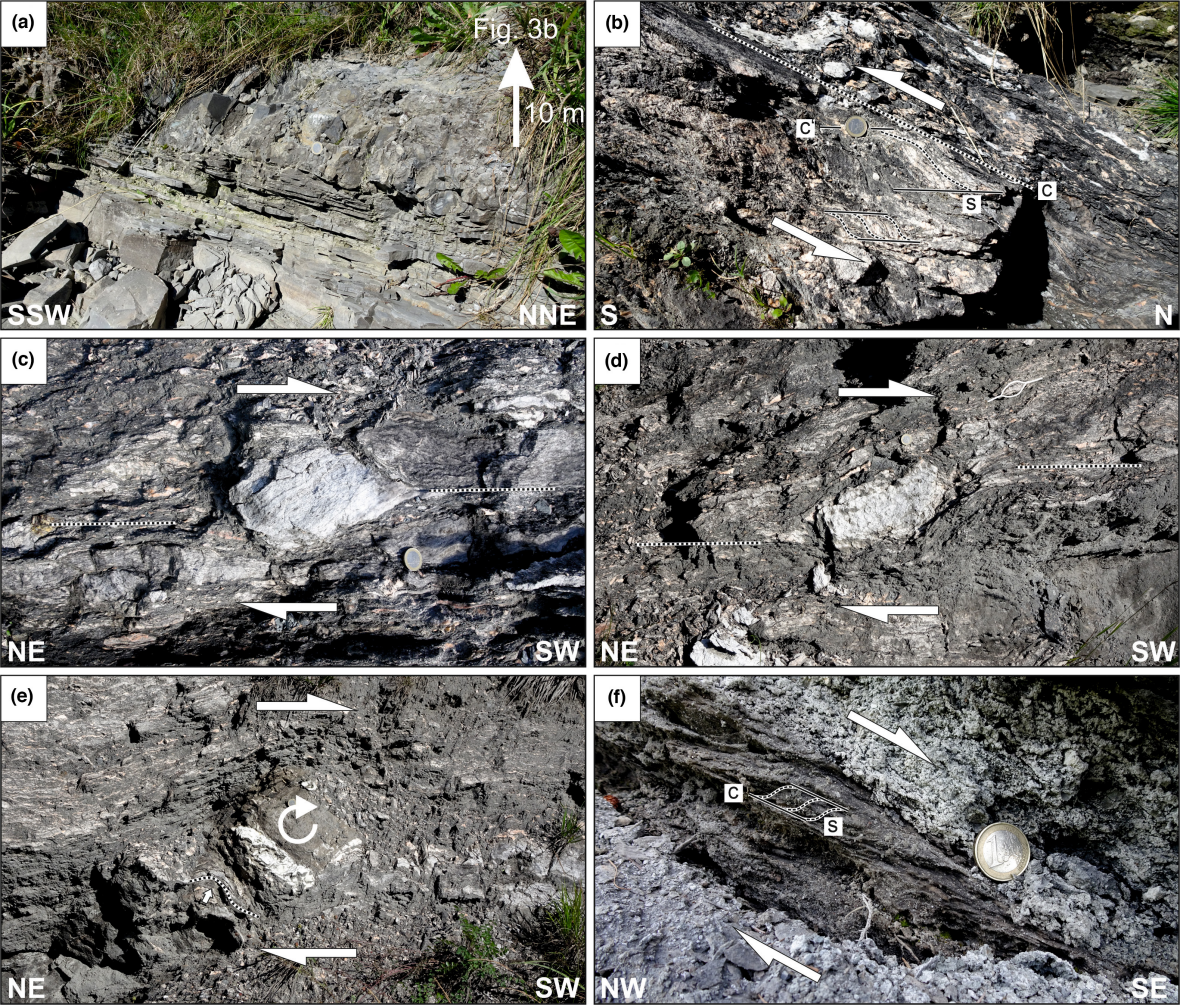
(a) Conglomerate layer with up to 15 cm large limestone components in the otherwise planar cm‐bedded siliceous limestone of the Ruhpolding Fm. This outcrop (UTM33 446336E, 5,274,432 N) is 6 metres stratigraphically below the Haselgebirge (Figure 3b). (b) Mylonitic Haselgebirge (UTM33 446333E, 5,274,444 N) with scc’ fabric indicating top‐to‐the south shear sense (c‐plane 350/38, stretching lineation on c‐plane 005/36). (c,d) Sigmoidal‐shaped anhydrite components stabilized with long axes inclined against the shear direction. Note, the stair stepping of the foliation in the Haselgebirge indicates top‐to‐the SW shear sense (UTM 33445676E, 5,274,675 N). (e) Rolling structure of an angular sandstone clast coupled with the foliation in the Haselgebirge (UTM33 445690E, 5,274,658 N). The clear embayment of the foliation in the Haselgebirge (grey arrow) suggests a co‐shearing rotation of the clast indicating top‐to‐the SW shear sense. (f) Sigmoidal secondary foliation s (305/08) in the Haselgebirge dips against the top‐to‐the SE shear sense on the c‐planes (114/33). The stretching lineation (126/32) on the c‐planes is roughly perpendicular to the intersection of the s‐ and c‐planes (UTM33 445288E, 5,276,731 N). Location of all figures is shown in Figure 1c.

Along its southern outcrop edge, the Haselgebirge Fm of the Wurzeralm rests directly on the Ruhpolding Fm. The contact between both units is masked by vegetation in outcrop (Figure 3a, Supplemental information S1), but geological mapping shows the surface dips under the Haselgebirge Fm, parallel to bedding of the underlying Ruhpolding Fm (Figure 1a, Supplemental information S2). Although the Ruhpolding Fm is undeformed and concordant with the underlying stratigraphy, the Haselgebirge presents a penetrative mylonitic fabric indicating south‐directed emplacement (Figure 3b). Isolated levels of Dachstein Limestone sedimentary breccias have been found within the Ruhpolding Fm under the salt sheet (Figure 3a).

The Haselgebirge Fm in the area is characterized by mylonitic fabric similar to that observed along its southern margin (3c‐f). Stretching lineations and shear sense indicators in the gypsum consistently show top‐to‐the‐south shearing (ranging between southwest and southeast) (Figure 1c). Along the southern outcrop border, the stretching direction is somewhat variable, recording non‐plane‐strain finite deformation (Figure 1c).

The Haselgebirge Fm is overlain mostly by the Werfen Fm and locally directly by the Allgäu and Ruhpolding Fms (Figure 1b). The Ruhpolding Fm is intensely folded, with fold axes trending in two perpendicular directions. One trends NE–SW (azimuth 020–070) and the other NW–SE (azimuth 300–320). The NE–SW fold trend is not observed in younger lithologies, nor does it coincide with the local shearing direction in the Haselgebirge Fm, and is therefore potentially related to soft‐sediment deformation above the Wurzeralm diapir during deposition (Supplemental Information S3). The NW–SE trend, in turn, is parallel to thrusts and folds involving the Oberalm and Haselgebirge Fms that potentially conditioned fabric development in the Haselgebrige Fm locally (Supplemental Information S3).

The Wurzeralm salt sheet is capped by the Plassen and Oberalm Fms. The reefal Plassen Limestone in the north transitions to the deeper water Oberalm Fm to the south. At present, the Plassen Limestone of the Wurzeralm lies at an elevation equivalent to that of the Dachstein Fm west of the Wurzeralm diapir due to activity of a post‐Jurassic eastward dipping normal fault (Figure 2d).

Along the western margin of the diapir, Rofan Breccia deposited along the edge of the evaporite body contain reworked limestone fragments of the Dachstein, Allgäu and Oberalm Fms, dark shale fragments that likely originate from the Haselgebirge Fm, and isolated clasts of Hallstatt Limestone (Ottner, 1990).

## 4 DISCUSSION

**FIGURE 4:**
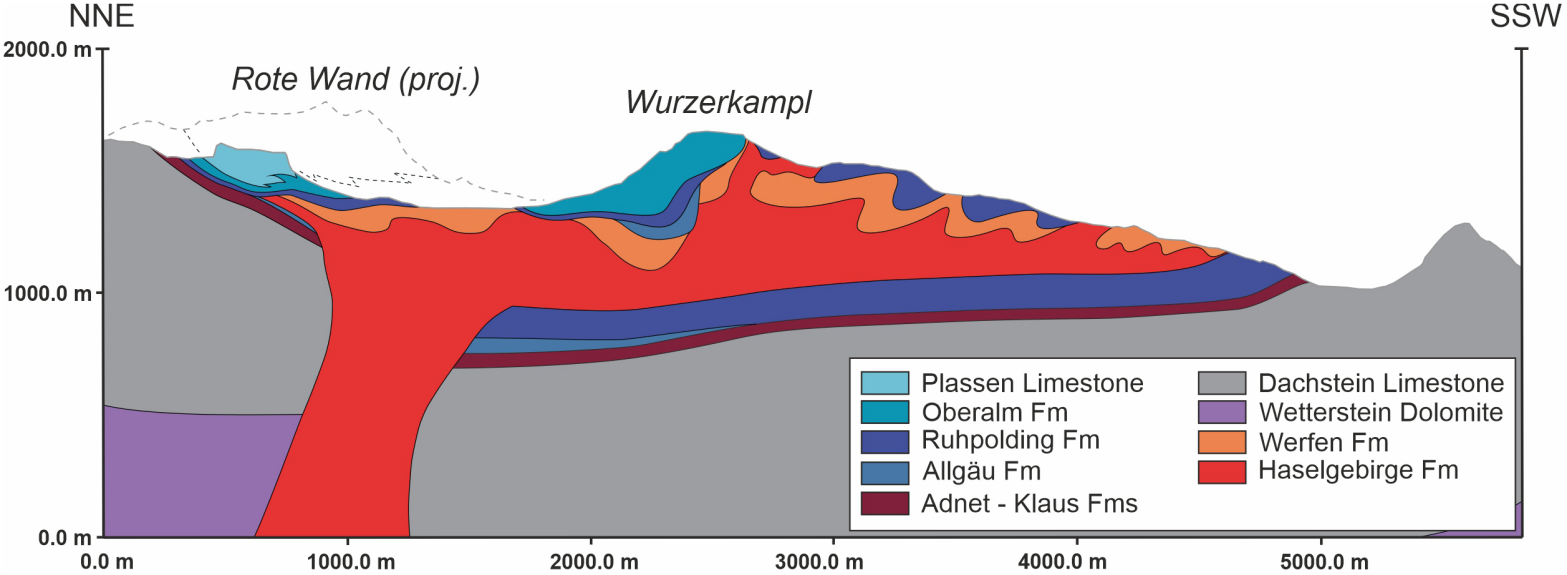
Structural cross‐section across the Wurzeralm salt allochthon. Structure at depth is inferred, including the regional thickness of the Dachstein Limestone derived from constraints beyond the study area.

**FIGURE 5:**
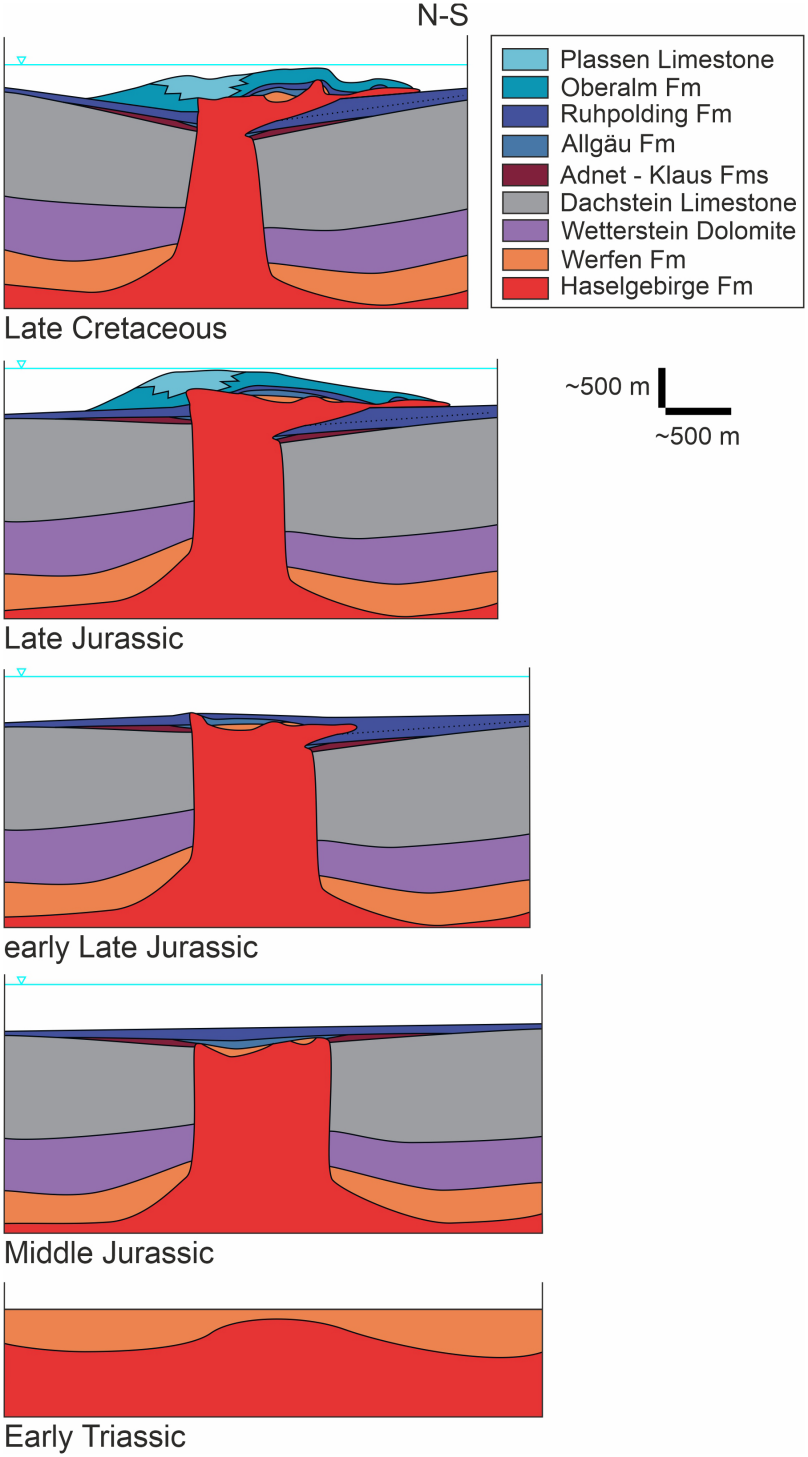
Conceptual evolutionary model of the Wurzeralm salt sheet. Sketch not to scale (approximate horizontal and vertical scales provided). The Triassic evolution of the structure was dominated by passive growth of a diapir and deposition of the Wetterstein and Dachstein Fms in flanking minibasins. This was followed by a period of quiescence and partial subsidence of the diapir during Early to Middle Jurassic. During this period, condensed red limestones (Adnet/Klaus Fms) deposited on the shallower minibasins flanking the diapir and the Allgäu Fm mainly over the subsiding diapir. This whole suite was draped over by the Ruhpolding Fm. Initial shortening in Late Jurassic times led to initial allochthony, synchronous with deposition of the youngest Ruhpolding Fm. As allochthony developed fully, both flanks of the diapir were tilted; the southern one downwards and the northern one upwards. The Upper Jurassic Plassen and Oberalm Fms deposited during this phase, with the shallower Plassen reef growing preferentially above the salt stock and the tilted northern flank of the diapir. Finally, during Late Cretaceous times, the salt structure collapsed partially to its almost present‐day structure.

The structure and outcrop distribution of the study area is interpreted to show the relict structure of a southward‐directed plug‐fed extrusion (Figure 4). Extrusion of the Haselgebirge Fm onto the seafloor above Middle Jurassic sediments occurred during the Late Jurassic (Figure 5) and was driven by the shortening of the diapiric salt stock. Based on the stretching lineations (Figure 1c), we interpret a dominantly southeast‐ to southwestward emplacement. Stretching perpendicular to the south‐directed flow along the southern front of the allochthon and at a location below the Ruhpolding Fm (Figure 1c) is non‐directional and likely due to transverse (circumferential) extension in the shallower portions of the allochthon (analogue to that modelled for axisymmetrical extrusive flow; Buisson & Merle, 2004; Závada et al., 2009).

We interpret that the salt stock that fed the salt sheet is located south of the Rote Wand (Figures 1a, 2a, and 4) given that (1) here the Werfen Fm outcrops lie deeper than the Middle Jurassic of the Rote Wand (i.e., in non‐allochthonous position) (Figure 2a); (2) this location is north of the northermost south‐directed shear indicator (Figure 1c); and (3) this area and the Rote Wand were the shallowest during the Late Jurassic (shallowest water facies; Figure 5) which could be explained by crestal doming above the salt stock during allochthony (e.g., Dooley et al., 2015) and upward tilting of the northern diapir flank (Figure 5). Uplift of this flank led to erosion of the Lower to Middle Jurassic under the Rote Wand (Figure 1a). The location of the southern wall of the salt stock is expected to be north of the northermost south‐directed shear indicator (Figure 1c). The width of the salt stock in Figure 4 is taken from the analogous Hallstatt diapir (Fernandez et al., 2021).

The present‐day structure suggests that the allochthonous salt sheet was at least 500 m thick (Figure 4), a magnitude compatible with dynamic topographies documented in plug‐fed extrusions (e.g., Dooley et al., 2015). The present‐day elongate asymmetric shape of the salt sheet and the absence of Upper Jurassic deposits beyond the faults, we name here Hintersteinerbach and Standseilbahn faults (Figure 1a), suggest that these faults bounded the salt sheet during emplacement. These faults are interpreted to cut across the entire Triassic stratigraphy, as indicated by the fact that a splay of the Hintersteinerbach juxtaposes the Dachstein Fm against the Wetterstein Fm (Figure 2a), and the panel of Triassic platform they bound may have been tilted down northwards during shortening (Figure 5), guiding the emplacement of the salt sheet. The northern extension of the Hintersteinerbach fault was later activated in extension (possibly during the Late Cretaceous, similar to structures documented further west by Fernandez et al., 2022), dropping the Jurassic Plassen Limestone down to the same elevation as the Dachstein Limestone (Figure 2a,d).

The link between deformation of the evaporites and that of the overlying units is not univocal. As discussed above, NE–SW trending folds in the Ruhpolding Fm likely relate to syn‐sedimentary deformation atop the diapir (Supplemental Information S3). Diapir‐related instability potentially also affected surrounding areas, as recorded by the presence of breccias in the sub‐allochthon Ruhpolding Fm (Figure 3a). Posteriorly, during emplacement, roof units were likely passively dismembered (e.g., Dooley et al., 2015), and partly eroded and accumulated in the Rofan Breccia bodies above and ahead of the open‐toe allochthon front. Notwithstanding, NW–SE trending folds and thrusts in the Ruhpolding and Oberalm Fm also involved deformation with the Haselgebirge Fm (Supplemental Information S3). These structures are tentatively related to constriction of the salt sheet between the Hintersteinerbach and Standseilbahn faults (Figure 1a).

Although the Wurzeralm structure has previously been explained as the result of gravitational northward sliding (Lein, 1987a; Ottner, 1990; Tollmann, 1987), the top‐to‐the‐south shear in the gypsum of the Haselgebirge Fm (Figure 3) rules out a south‐derived origin for the Wurzeralm salt (either by sliding or by thrusting). Southward emplacement of the salt sheet can be accounted for by an episode of Jurassic compression‐driven allochthony (Figure 5). The mylonitic fabric likely developed through pressure solution‐precipitation creep (e.g., Závada et al., 2021) and required rates of deformation that are more compatible with gradual allochthony than with near instantaneous gravitational sliding. Furthermore, a similar history of Triassic diapir growth and Jurassic shortening, as opposed emplacement of the Haselgebirge evaporites by gravitational sliding, has been documented by Fernandez et al. (2021) in the Hallstatt diapir, some 50 km to the west. The Wurzeralm is in line with a growing body of examples recently highlighting the relevance of salt tectonics in the NCA Granado et al. (2019) and Strauss et al. (2021).

Schorn and Neubauer (2011) also documented mylonitic fabric in Haselgebirge Fm anhydrite in the Moosegg body (some 100 km to the west), but interpreted it as necessarily resulting from thrusting. Our observations and those of Závada et al. (2021) indicate that mylonitic fabric in anhydrite can develop at shallow levels, making it possible to propose a possible alternative origin for the Moosegg body as an allochthon. The capping of the Moosegg body by Lower Cretaceous clastics and the presence of fragments of Haselgebirge within the Lower Cretaceous sediments surrounding it (Schorn & Neubauer, 2011) could be explained by surface exposure of the Haselgebirge Fm during allochthony. An equivalent situation is that of the Bad Ischl salt accumulation, which rests on Lower Cretaceous sediments (Mandl et al., 2012). MORB‐affinity igneous rocks found within both of these bodies (Schorn et al., 2013; Vozarova et al., 1999), not expected in diapirs soled above continental crust, are common components of Lower Cretaceous clastics (Krische et al., 2014) and might have been incorporated during emplacement.

Likewise, occurrences of Haselgebrige Fm on a Middle Jurassic seafloor documented at other locations in the NCA (e.g., Mandl, 2016; Plöchinger, 1996; Suzuki & Gawlick, 2009) might be candidates to evaluate as the possible result of salt allochthony.

The Wurzeralm also provides important insights into the Jurassic bathymetric evolution of the area. It is generally accepted that the NCA deepened during the Early to Middle Jurassic culminating during deposition of the Ruhpolding Fm (Lein, 1987b; Mandl, 2000). Uplift of the seafloor into the photic zone during the Late Jurassic was necessary for the growth of Plassen Limestone reefs. Plassen reefs occur in the NCA mostly as isolated reefs, often above Haselgebirge Fm accumulations (Tollmann, 1976, 1987). Uplift driven by doming of salt above its stock during extrusion (Figure 5) can account for the isolated nature of reef development, with the crestal bulge being the only point to bathymetrically rise into the photic zone. For this to be possible, assuming up to 1000 m of salt‐driven uplift (Dooley et al., 2015), the Ruhpolding Fm must have deposited in water not much deeper than 1000 m (compatible with the depth of other Tethyan Jurassic radiolarites; Baumgartner, 2013). In the absence of regional uplift, sedimentation during the Late Jurassic would have focused around the developing reef, as indicated by the absence of Upper Jurassic sediments to the west and north of the Wurzeralm (Figure 1a; Moser & Pavlik, 2014) and by their greatly reduced thickness further to the east (Tollmann, 1976).

## 5 CONCLUSION

Despite the magnitude of Alpine overprint in the area, the Jurassic‐age Wurzeralm salt sheet presents an almost intact structure and is a striking example of a submarine‐emplaced allochthonous salt body with syn‐emplacement roof sedimentation. This salt sheet constitutes the first instance of a preserved diapir and its associated allochthon documented in the Eastern Alps, underscores the relevance that salt tectonics played in this area and highlights the need of revisiting the interpretation of other salt bodies in the Eastern Alps.

## Supporting information


Data S1

## Data Availability

The data that support the findings of this study are available from the corresponding author upon reasonable request.
