# Supplementary material for: Emplacement and associated sedimentary record of the Jurassic submarine salt allochthon of the Wurzeralm (Eastern Alps, Austria)
Source: Terra Nova. 2023 Aug 7;35(6):524–32. doi: 10.1111/ter.12675 (PMC10958983; doi:10.1111/ter.12675)
Supplement: Supplementary file 1 — Data S1 [file TER-35-524-s001.pdf]

Supplemental information 1: 3D outcrop of basal allochthon contact

The 3D model below has been generated from oblique photographs taken with a handheld digital Panasonic Lumix DMC-FZ1000 II camera with a 20 Megapixel CMOS-1" sensor. Images have been processed with a Structure from Motion algorithm (Agisoft Metashape Professional v. 1.8.3, www.agisoft.com) to generate a point cloud with 24,469,626 points and a textured mesh with 1,408,843 faces. The model has been georeferenced and scaled based on outcrop control points.

In the model the location of Fig. 3a and 3b are shown by the blue and dark orange spheres respectively (the blue sphere lies beyond the southern extent of the mesh). The yellow sphere indicates the location of the top of the Jurassic Ruhpolding Fm siliceous limestones and base of the allochthonous evaporite unit. The lowermost 3 meters of the evaporite unit are not directly visible and are covered by mud.

Supplemental information 2: 3D model of topography and dip data

The 3D model of the Wurzeralm study area includes the downsampled topography (Geoland.at, 2015) draped with the geological map of Figure 1a and a selection of dip measurements manually filtered for representativity, to avoid clutter. Dip data (represented by orientated disks) are derived from either bedding dip measurements acquired by the co-authors in the field or from analysis of digital topography and 3D digital outcrop data, following the approach described in Fernandez et al. (2009).

Note that the basal contact of the Wurzeralm allochthonous salt body can be traced around the outcrop of the allochthon, with dips of the underlying Ruhpolding Fm indicating this unit dips under the allochthonous Haselgebirge and Werfen Fms. Furthermore, the basal contact of the allochthon strikes roughly parallel to the stratigraphic contact between the Ruhpolding Fm and the underlying Klaus Fm or Dachstein Limestone, indicating the contact has a similar dip and trend (i.e., roughly parallel to bedding).

Supplemental information 3: Folding in the Ruhpolding Fm

Folding in the Ruhpolding Fm is found to follow two main trends roughly perpendicular to each other: NE-SW and NW-SE.

SI.3a: Location of selected detailed observations of deformation involving the Ruhploding Fm in the study area. This map and its color legend are equivalent to those in Figure 1c.

SI.3b: Outcrop view (from UTM33 446210E 5276624N) trending roughly E-W (slope trends NE-SW) of an imbricate thrust system causing a repetition of the Oberalm, Ruhpolding and Haselgebirge Fms. The structures trend roughly NW-SE. Note that the stretching lineation is parallel to the trend of the folds and faults in the Jurassic units, with a broad scatter of foliation planes in the Haselgebirge Fm, raising the possibility that the fabric is nearly a purely constrictional fabric associated to folding-thrusting (cf. Fig. 8, Sullivan, 2013). Thrusting and

folding are considered to be posterior to Ruhpolding Fm consolidation, as they involve the entire Oberalm Fm as well. The origin is therfore either tectonic or related to salt-sheet emplacement.

SI.3c,d: Drone-acquired aerial oblique views of an outcrop (UTM33 446105E 5276624N) exhibiting complex folding within the Ruhpolding Fm. A common point on both photos is indicated by a black line joining them. Fold axes (measured, as well as best-fit calculations) have a NE-SW trend. The outcrop exhibits a chaotic structure in its eastern segment (SI.3d) and partially distorted beds in the western segment, recognized by the more nodular geometry in the core of the main fold structure (SI.3c). This is interpreted to result from syn-sedimentary to shallow-burial deformation. The main fold structure of the western segment (SI.3c) shows constant-thickness bedding and geometries reminiscent of post-burial (consolidated) state deformation (M crestal fold). However, given the limited amount of maximum burial of the area (e.g., Kralik et al., 1987; Gawlick et al., 1994), the tightness of folding is easier to explain assuming the radiolarite unit was not fully consolidated. The dimensions of these folds (tens of meters) are compatible to those of other known soft-sediment deformation folds (e.g., Ortner, 2007).

SI.3e: Outcrop view (UTM33 446115E 5276815N) trending roughly N-S of Ruhpolding Fm beds affected by folding with NW-SE trending fold axes that overprint initial folding and truncation of beds related to syn-sedimentary deformation. In this outcrop two fold axes have been estimated. One fold axis has been estimated from beds that have been steepened by the NW-SE trending fold and coincides with a minor fold axis measured in the field (outside the field of the photo). The second fold axis has been determined for beds that appear truncated (as these clearly relate to syn-sedimentary deformation). Although the best-fit calculation in this case is based on little data, it indicates in any case a fold axis that trends at a significantly high angle to the first axis, and that plunges roughly parallel to bedding (as would be expected for a syn-sedimentary fold).

References

Fernandez, O., Jones, S., Armstrong, N., Johnson, G., Ravaglia, A., Muñoz, J.A., 2009. Automated tools within workflows for 3D structural construction from surface and subsurface data. *Geoinformatica*, **13**, 291-304. doi: 10.1007/s10707-008-0059-y

Gawlick, H.-J., Krystyn, L., Lein, R., 1994. Conodont colour alteration indices: Palaeotemperatures and metamorphism in the Northern Calcareous Alps - a general view. *Geologische Rundschau*, **83**, 660-664.

Geoland.at, 2015. Digital terrain model of Austria, based on airborne laserscanning. Geoland.at. https://www.data.gv.at/katalog/de/dataset/dgm

Kralik, M., Krumm, H., Schramm, J.M., 1987. Low grade and very low grade metamorphism in the Northern Alps and in the Grauwacke Zone: Illite-crystallinity data and isotopic ages. In: *Geodynamics of the Eastern Alps* (H.W. Flügel, P. Faupl, eds.), Franz Deuticke, 164-178.

Ortner, H., 2007. Styles of soft-sediment deformation on top of a growing fold system in the Gosau Group at Muttekopf, Northern Calcareous Alps, Austria: Slumping versus tectonic deformation. *Sedimentary Geology*, **196**, 99-118. doi: 10.1016/j.sedgeo.2006.05.028

Sullivan, W.A., 2013. L tectonites. *Journal of Structural Geology*, **50**, 161-175.

SI.1. 3D outcrop of basal allochthon contact

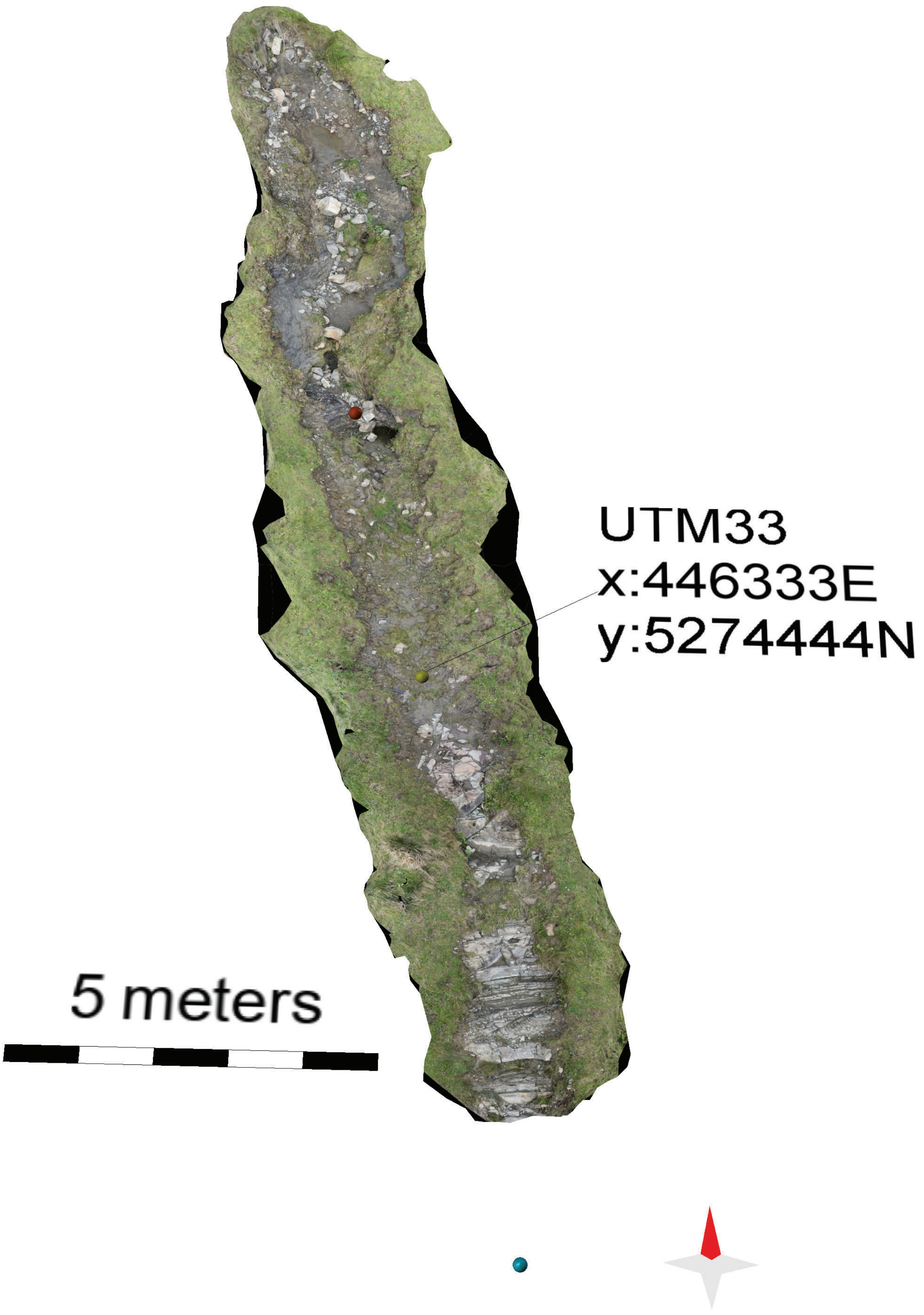

Created using move™

SI.2. 3D model of topography and dip data

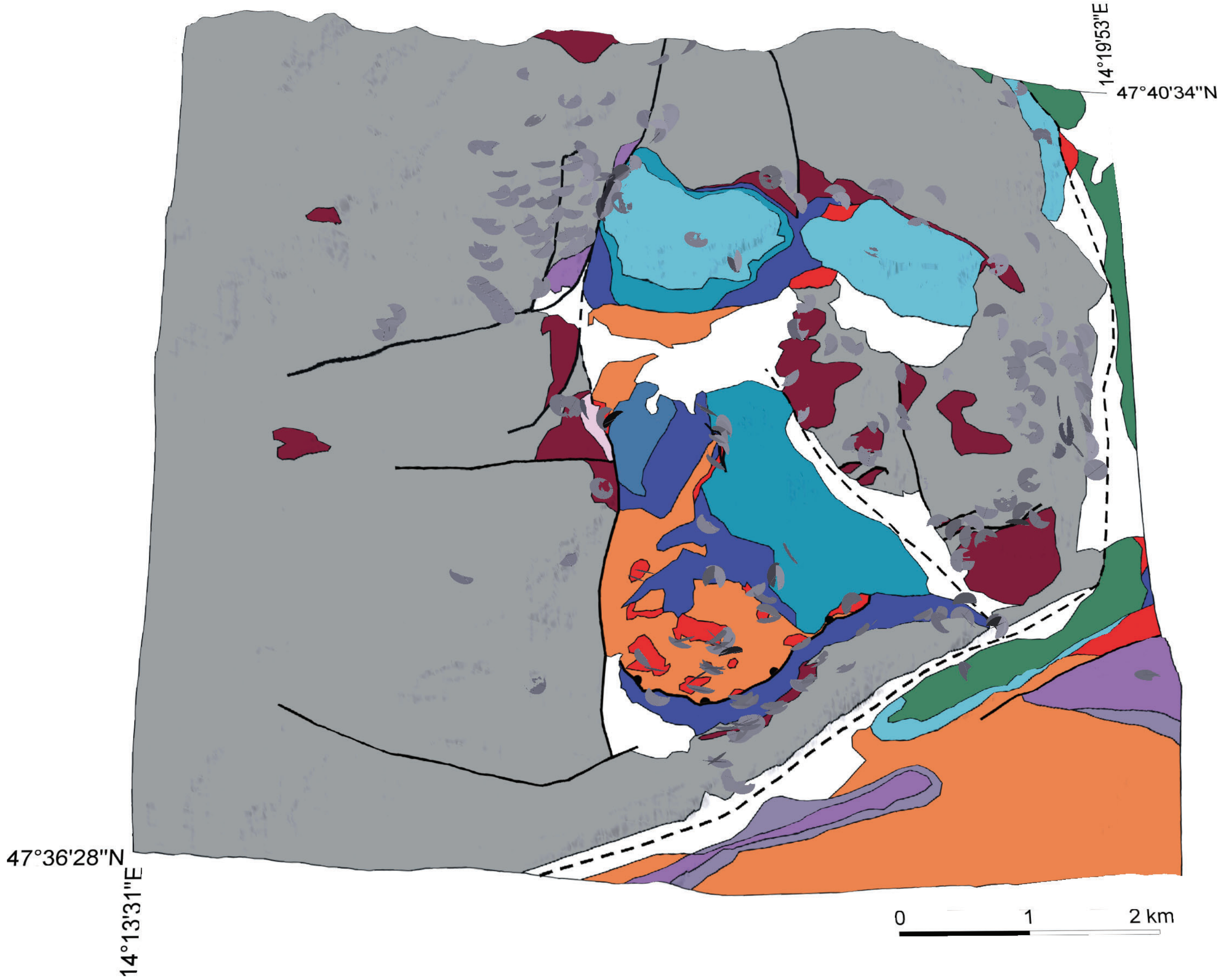

Petroleum Experts
Created using move™

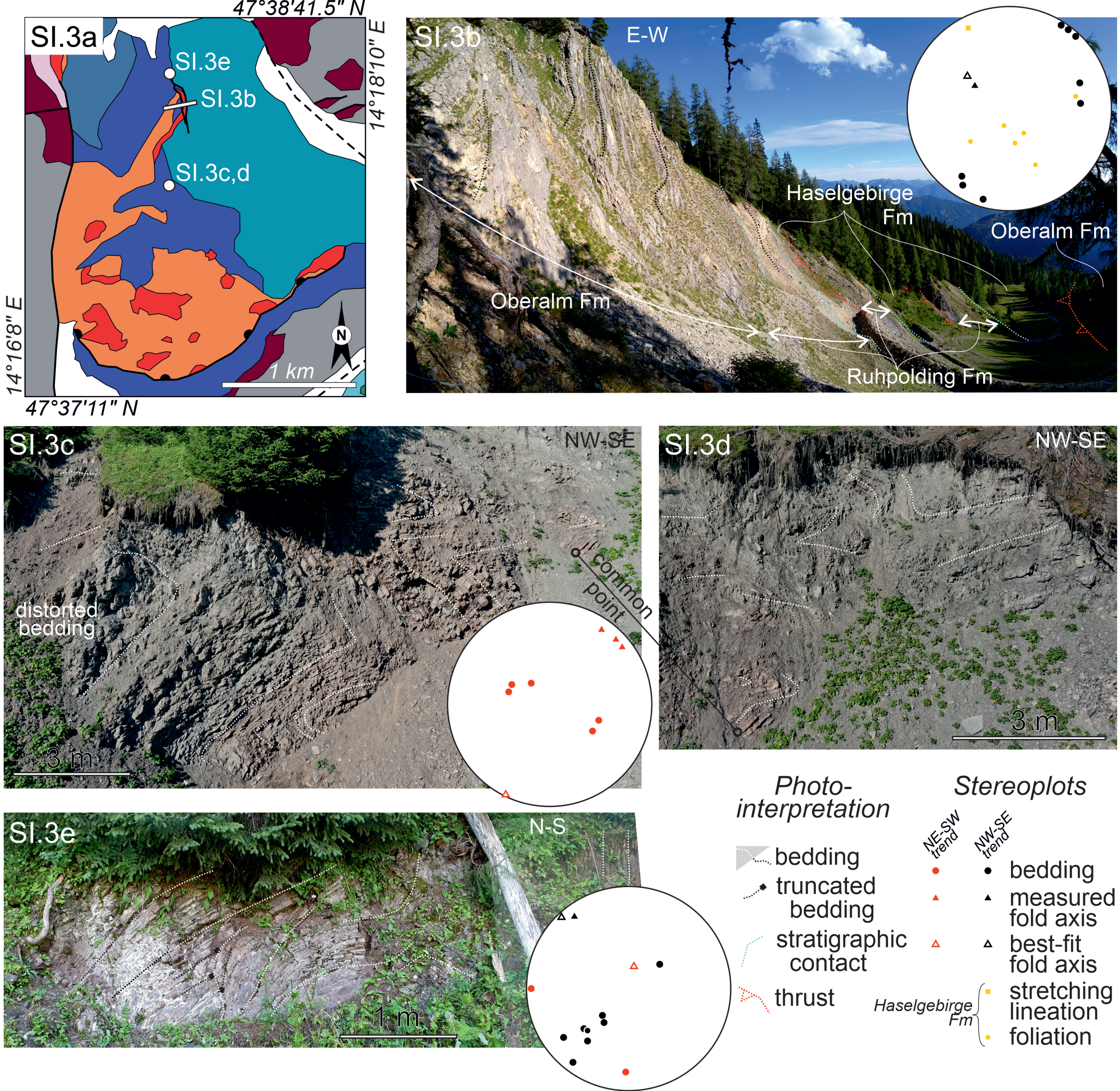
SI.3a
47°38'41.5" N
14°18'10" E
14°16'8" E
47°37'11" N
SI.3e
SI.3b
SI.3c,d
N
1 km
SI.3b
E-W
Haselgebirge Fm
Oberalm Fm
Oberalm Fm
Ruhpolding Fm
SI.3c
NW-SE
distorted bedding
common point
3 m
SI.3d
NW-SE
3 m
SI.3e
N-S
1 m
Photo-interpretation
bedding
truncated bedding
stratigraphic contact
thrust
Stereoplots
NE-SW trend
NW-SE trend
bedding
measured fold axis
best-fit fold axis
Haselgebirge Fm
stretching lineation
foliation
